# Selective catalytic reduction of NO*_x_* with NH_3_: opportunities and challenges of Cu-based small-pore zeolites

**DOI:** 10.1093/nsr/nwab010

**Published:** 2021-01-16

**Authors:** Yulong Shan, Jinpeng Du, Yan Zhang, Wenpo Shan, Xiaoyan Shi, Yunbo Yu, Runduo Zhang, Xiangju Meng, Feng-Shou Xiao, Hong He

**Affiliations:** State Key Joint Laboratory of Environment Simulation and Pollution Control, Research Center for Eco-Environmental Sciences, Chinese Academy of Sciences, Beijing 100085, China; State Key Joint Laboratory of Environment Simulation and Pollution Control, Research Center for Eco-Environmental Sciences, Chinese Academy of Sciences, Beijing 100085, China; College of Resources and Environment, University of Chinese Academy of Sciences, Beijing 100049, China; Center for Excellence in Regional Atmospheric Environment, Institute of Urban Environment, Chinese Academy of Sciences, Xiamen 361021, China; Center for Excellence in Regional Atmospheric Environment, Institute of Urban Environment, Chinese Academy of Sciences, Xiamen 361021, China; State Key Joint Laboratory of Environment Simulation and Pollution Control, Research Center for Eco-Environmental Sciences, Chinese Academy of Sciences, Beijing 100085, China; College of Resources and Environment, University of Chinese Academy of Sciences, Beijing 100049, China; State Key Joint Laboratory of Environment Simulation and Pollution Control, Research Center for Eco-Environmental Sciences, Chinese Academy of Sciences, Beijing 100085, China; Center for Excellence in Regional Atmospheric Environment, Institute of Urban Environment, Chinese Academy of Sciences, Xiamen 361021, China; College of Resources and Environment, University of Chinese Academy of Sciences, Beijing 100049, China; State Key Laboratory of Chemical Resource Engineering, Beijing Key Laboratory of Energy Environmental Catalysis, Beijing University of Chemical Technology, Beijing 100029, China; Key Laboratory of Applied Chemistry of Zhejiang Province, Department of Chemistry, Zhejiang University, Hangzhou 310007, China; Key Laboratory of Applied Chemistry of Zhejiang Province, Department of Chemistry, Zhejiang University, Hangzhou 310007, China; State Key Joint Laboratory of Environment Simulation and Pollution Control, Research Center for Eco-Environmental Sciences, Chinese Academy of Sciences, Beijing 100085, China; Center for Excellence in Regional Atmospheric Environment, Institute of Urban Environment, Chinese Academy of Sciences, Xiamen 361021, China; College of Resources and Environment, University of Chinese Academy of Sciences, Beijing 100049, China

**Keywords:** environmental catalysis, Cu-SSZ-13, NH_3_-SCR, reaction mechanism, small-pore zeolites

## Abstract

Zeolites, as efficient and stable catalysts, are widely used in the environmental catalysis field. Typically, Cu-SSZ-13 with small-pore structure shows excellent catalytic activity for selective catalytic reduction of NO*_x_* with ammonia (NH_3_-SCR) as well as high hydrothermal stability. This review summarizes major advances in Cu-SSZ-13 applied to the NH_3_-SCR reaction, including the state of copper species, standard and fast SCR reaction mechanism, hydrothermal deactivation mechanism, poisoning resistance and synthetic methodology. The review gives a valuable summary of new insights into the matching between SCR catalyst design principles and the characteristics of Cu^2+^-exchanged zeolitic catalysts, highlighting the significant opportunity presented by zeolite-based catalysts. Principles for designing zeolites with excellent NH_3_-SCR performance and hydrothermal stability are proposed. On the basis of these principles, more hydrothermally stable Cu-AEI and Cu-LTA zeolites are elaborated as well as other alternative zeolites applied to NH_3_-SCR. Finally, we call attention to the challenges facing Cu-based small-pore zeolites that still need to be addressed.

## Teaser

This review article summarizes the major advances, opportunities and challenges of Cu-based small-pore zeolites applied to environmental catalysis, which provides guidance for the design of highly efficient and stable zeolite-based catalysts.

## INTRODUCTION

### General introduction to zeolite catalysts, NO*_x_* pollution and SCR technology

Zeolites are a group of crystalline inorganic materials with regular pore structures that consist of connected TO_4_ tetrahedra (T represents the framework atom) sharing oxygen atoms. Generally, the zeolites can be divided into small-pore zeolites with 8-membered rings (pore size of ∼4.0 Å), medium-pore zeolites with 10-membered rings (pore size of ∼5.5 Å), large-pore zeolites with 12-membered rings (pore size of ∼7.5 Å) and ultra-large-pore zeolites with >12-membered rings. The pore size of zeolites is similar to molecular sizes, which endows the zeolites with a molecular sieving effect, resulting in excellent shape selectivity. Their inner channel and cavity space give zeolites huge specific surface areas. Zeolite materials also have high hydrothermal stability due to their highly crystalline framework structure. Moreover, the abundant acid sites of zeolites can be easily adjusted by ion-exchange methods to satisfy the conditions of various chemical reactions. Based on these advantages, therefore, zeolites are widely used in the petrochemical, energy and environmental fields as efficient and stable catalysts [1–3]. An atomic-scale understanding of zeolite applications in different fields is of guiding significance to the design and synthesis of zeolite catalysts. The present work focuses on the field of environmental catalysis, in which zeolite catalysts play an indispensable role.

In the environmental field, nitrogen oxides (NO*_x_*, including NO and NO_2_) are important precursor pollutants for haze, photochemical smog and acid rain. In China, transportation contributes ∼35% of NO*_x_* emissions, second only to industry (∼40%) as an emissions source [4]. Diesel vehicles, which account for about 10% of automobiles, produce nearly 90% of NO*_x_* (total ∼6.4 million tonnes) emitted from automobiles [5]. In addition, non-road mobile sources, which primarily use diesel engines as a power source and include engineering machinery, farming machinery and marine engines, contributed comparable NO*_x_* emissions to those of road automobiles in China. Therefore, controlling the NO*_x_* pollutants emitted from diesel engines is urgent and imperative. The SuperTruck Program and Horizon 2020 were launched by the United States and the European Union to increase engine efficiency and reduce pollutants. China will also implement the China VI Standards for emissions from diesel-fuelled heavy-duty vehicles. In the aftertreatment system, the three-way catalyst (TWC) has difficulty controlling NO*_x_* emissions due to the lean combustion conditions in diesel engines. For controlling diesel vehicle exhaust, the selective catalytic reduction of NO*_x_* with ammonia (NH_3_-SCR) has been successfully and commercially applied because of its high NO*_x_* efficiency in the presence of excess oxygen.

**Figure 1.**
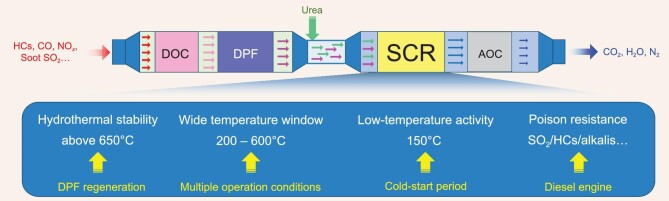
DOC + DPF + SCR + AOC aftertreatment system of diesel vehicles.

To meet progressively more rigorous legislations and policies, a complicated aftertreatment system was proposed and employed comprising multiple processing units (Fig. 1), in which the SCR unit is located in a downstream position. The DOC (diesel oxidation catalyst) is used to oxidize the hydrocarbons (HCs) and CO and partially oxidize NO to NO_2_ with excess oxygen. The partial oxidation of NO benefits the fast SCR reaction, which requires an equimolar mixture of NO and NO_2_. PMs (particulate matters) are captured and filtered by the DPF (diesel particulate filter). The reason for the placement of the AOC (ammonia oxidation catalyst) is to eliminate NH_3_ slip from the SCR unit. As can be seen, the SCR catalyst must have excellent hydrothermal stability due to the high-temperature environment (above 650°C) resulting from regeneration of the upstream DPF. Additionally, the complicated and changeable operation conditions of diesel vehicles expose the SCR catalyst to a wide variety of temperatures (200–600°C), including low temperatures below 200°C under cold-start and low-load conditions. Catalyst poisoning is also inevitable due to the incomplete oxidation of HCs and CO as well as the presence of S- and alkali-metal-containing diesel fuels. Therefore, SCR catalysts should be evaluated comprehensively and satisfy various operating modes in actual application. Fortunately, the zeolite catalysts are well-qualified as SCR catalysts due to their outstanding deNO*_x_* efficiency, high hydrothermal stability and optimal poison resistance.

### NH_3_-SCR reaction features and NH_3_-SCR catalysts

The NO*_x_* emitted from diesel engines consists of 90% NO and 10% NO_2_. Therefore, the primary reaction of NH_3_-SCR is the standard SCR reaction (SSCR, reaction (1)). In this redox reaction, 12 electrons are transferred. The N (−3 valence) in NH_3_ transfers 8e^−^ and 4e^−^ to NO and O_2_, respectively. Oxygen participates in the oxidation of active sites and combines with hydrogen to form H_2_O. Fast SCR (FSCR, reaction (2)), involving equal amounts of NO and NO_2_, also occurs when NO is partially oxidized in the DOC unit. Through this reaction, higher NO*_x_* conversion can usually be achieved, in which two and four electrons are transferred from NH_3_ to NO and NO_2_, respectively. Besides the standard and fast SCR reactions, there are also several side reactions (reactions (3–6)):

(1) \begin{equation*}4{\rm{N}}{{\rm{H}}_3} + 4{\rm{NO}} + {{\rm{O}}_2} \to 4{{\rm{N}}_2} + 6{{\rm{H}}_2}{\rm{O}}\end{equation*}

(2) \begin{equation*}2{\rm{N}}{{\rm{H}}_3} + {\rm{NO}} + {\rm{N}}{{\rm{O}}_2} \to 2{{\rm{N}}_2} + 3{{\rm{H}}_2}{\rm{O}}\end{equation*}

(3) \begin{equation*}6{\rm{N}}{{\rm{O}}_2} + 8{\rm{N}}{{\rm{H}}_3} \to 7{{\rm{N}}_2} + 12{{\rm{H}}_2}{\rm{O}}\end{equation*}

(4) \begin{equation*}4{\rm{N}}{{\rm{H}}_3} + 3{{\rm{O}}_2} \to 2{{\rm{N}}_2} + 6{{\rm{H}}_2}{\rm{O}}\end{equation*}

(5) \begin{equation*}2{\rm{N}}{{\rm{H}}_3} + 2{{\rm{O}}_2} \to {{\rm{N}}_2}{\rm{O}} + 3{{\rm{H}}_2}{\rm{O}}\end{equation*}

(6) \begin{equation*}4{\rm{N}}{{\rm{H}}_3} + 4{\rm{NO}} + 3{{\rm{O}}_2} \to 4{{\rm{N}}_2}{\rm{O}} + 6{{\rm{H}}_2}{\rm{O}}\end{equation*}

The NH_3_-SCR reaction needs the participation of both NO*_x_* (acid oxide) and NH_3_ (base). Therefore, dual-functional sites are of significant importance in the SCR reaction due to the double-cycle mechanism, including the redox cycle (NO*_x_*) and acid cycle (NH_3_). In addition, the tight coupling of redox and acid sites (redox-acid sites) is beneficial in taking advantage of the synergistic effects of dual-sites [6,7]. For example, Fe-Ti and Ce-W oxide catalysts were developed and showed excellent NH_3_-SCR performance. The tight coupling of Fe (Ce) and Ti (W) is achieved by the co-precipitation method [7]. From another aspect, high dispersion and adequate exposure of functional sites, which increase the effective collision probabilities between active centres and reactants, are indispensable in every catalytic reaction.

Based on the above design principles, a series of redox-acid oxides with highly dispersed active sites were developed as novel and highly efficient NH_3_-SCR catalysts [7,8]. However, the thermal stability of oxide catalysts still needs to be improved due to the phase separation of the tight coupling structure of redox and acid sites at high temperatures. Since the Cu-SSZ-13 small-pore zeolite was reported to have superior activity and hydrothermal stability in the NH_3_-SCR reaction compared with medium- and large-pore zeolites (patent in 2009) [9–11], studies on Cu-SSZ-13 showed a dramatic increase in the past decade. The small-pore structure is primarily responsible for the high stability of the catalyst framework and active sites in Cu-SSZ-13 [11]. Concomitantly, other zeolites with similar small-pore structures, such as LTA, AEI, KFI, and RTH, also received much attention due to their comparable NH_3_-SCR performance and/or hydrothermal stability to Cu-SSZ-13. In this work, the selective catalytic reduction of NO*_x_* using the Cu-SSZ-13 zeolite catalyst is comprehensively reviewed with critical emphasis on the state of active sites, SCR reaction mechanism and synthetic methods, as well as poisoning resistance mechanisms (SO_2_, PO_4_^3−^, HCs, alkali and alkaline earth metals). Based on the research methodology employed for Cu-SSZ-13, other Cu-exchanged small-pore zeolites are described in detail. Great efforts have been made to achieve matching between the characteristics of Cu^2+^-exchanged small-pore zeolite and highly efficient NH_3_-SCR catalysts, which is of great importance for the design of high-efficiency zeolitic catalysts. Furthermore, we propose basic rules for designing a zeolite catalyst for the NH_3_-SCR reaction as well as future efforts in this research field. We are devoted to providing an easy-to-read and systematic review of SCR of NO*_x_* using Cu^2+^-exchanged small-pore zeolites as catalysts and to highlighting the opportunities and challenges of Cu-based small-pore zeolites.

### Cu-SSZ-13: A MODEL CASE FOR Cu^2+^-EXCHANGED ZEOLITE CATALYSTS FOR NH_3_-SCR

**Figure 2.**
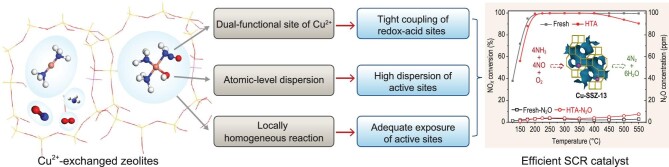
Matching between the characteristics of Cu^2+^-exchanged zeolitic catalyst and SCR catalyst requirements. The monolithic catalyst (∼2.36 cm^3^) was estimated to have a total gas flow of 1.67 L/min, resulting in GHSV of ∼42 000 h^−1^. HTA represents that the sample was hydrothermally aged at 750°C for 16 h.

As we discussed above, the highly efficient NH_3_-SCR catalysts contain tightly coupled redox-acid sites as well as high dispersion and adequate exposure of these functional sites. Remarkably, the characteristics of copper ion-exchanged zeolites match perfectly with NH_3_-SCR catalyst requirements, as shown in Fig. 2. The Cu^2+^/Cu^+^ couple can act as both redox and acid site in the NH_3_-SCR reaction due to its multi-valence state and Lewis acid characteristics, where the redox cycle (NO*_x_*) and acid cycle can both be completed; moreover, the Cu^2+^ ions in ion-exchanged zeolites are atomically dispersed, since the metal ions compensate the positive charge of the zeolite framework by anchoring on the ion-exchange sites. More importantly, the locally homogeneous reactions in the bulk phase, resulting from the dynamic NH_3_-solvated Cu ions and electronic effects of the zeolite, significantly increase the effective collision probability between reactants and active species [12]. Therefore, Cu^2+^-exchanged zeolites achieve the required attributes of tight coupling of redox-acid sites, high dispersion and adequate exposure of functional sites. In particular, small-pore zeolites such as Cu-SSZ-13 possess better NH_3_-SCR performance and higher hydrothermal stability, N_2_ selectivity and poisoning tolerance than other zeolites due to the peculiar small-pore structure. For example, the small pores (∼4.0 Å) of Cu-based small-pore zeolites inhibit dealumination of the framework and accumulation of copper species during hydrothermal aging, which is highly beneficial for hydrothermal stability [11]. Also, some poisons, such as long-chain hydrocarbons, are prevented from entering the small pores due to their shape selectivity. The cavities of small-pore zeolites offer abundant active sites and large reaction zones, thus facilitating the chemical reactions. Therefore, the NH_3_-SCR reaction requirements match perfectly with the physicochemical properties of Cu^2+^-exchanged zeolites (especially small-pore zeolites), providing a solid foundation for highly efficient and stable NH_3_-SCR zeolite catalysts.

**Figure 3.**
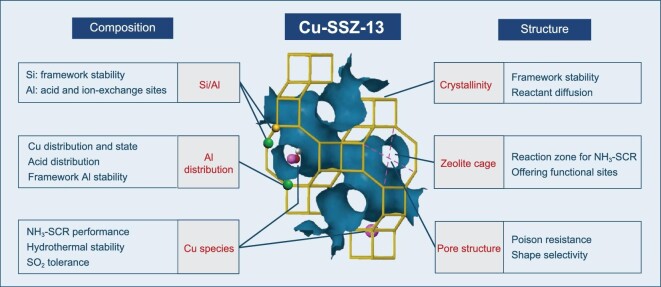
The composition-structure-activity (stability) relationship of Cu-zeolites for NH_3_-SCR reaction.

Due to the excellent deNO*_x_* efficiency and hydrothermal stability as well as the simplicity of the framework structure (only one T site), Cu-SSZ-13 zeolite, with chabazite (CHA) topology, is studied as a model case for understanding the composition-structure-activity (and stability) relationship of Cu^2+^-exchanged zeolite catalysts applied for NH_3_-SCR. As shown in Fig. 3, the CHA structure is constructed by stacking double six-membered rings (D6R) in an ABC sequence, resulting in a large CHA cavity and eight-membered ring (8MR) pore structure. The Si/Al ratio is of vital importance for the performance of Cu-SSZ-13 zeolite as an SCR catalyst. The large amount of Si in the substrate assures the stability of the zeolite framework, and the substitution of Si by Al provides acid sites due to charge compensation, which further benefits the location of active metal ions during the ion-exchange process and NH_3_ adsorption during the NH_3_-SCR reaction. Consequently, the Cu and acid site distribution as well as its stability are determined by the Al distribution in Cu-SSZ-13 zeolites. The state and location of Cu species directly affect the NH_3_-SCR performance, hydrothermal stability and SO_2_ tolerance of Cu-exchanged zeolites. As for the zeolite structure, the high crystallinity of zeolite favours framework stability and reactant diffusion. Reaction zones for NH_3_-SCR and adequate functional sites can be offered by the large zeolite cage (such as the CHA cavity). Importantly, the pore structure endows Cu-exchanged zeolites with shape selectivity catalysis, leading to high poison resistance, especially regarding some large molecules. After describing the general characteristics of Cu-SSZ-13, the application of Cu-SSZ-13 in the NH_3_-SCR reaction is elaborated in detail below.

### Copper species

**Figure 4.**
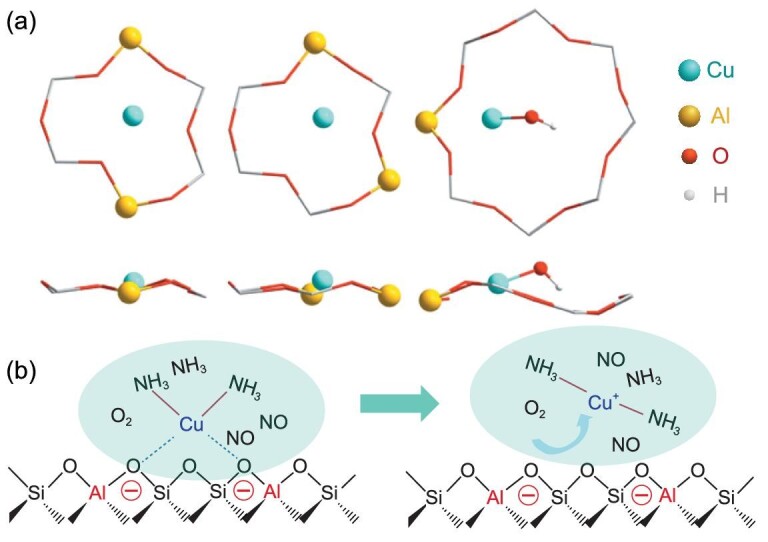
(a) Density functional theory (DFT) calculations of Cu^2+^ location and coordination. Adapted with permission from ref. [16]. (b) The NH_3_ solvation effect of Cu^2+^ and dynamic Cu-(NH_3_)_n_ complex during SCR reaction.

The state and location of copper species are closely related to the elemental composition of the zeolite and the atmospheric environment. The substitution of Si atoms by Al atoms creates charge defects, which provide Brønsted acid sites and exchange sites for Cu ions. The isolated Cu^2+^ ions were initially thought to be the active centres and to be coordinated to three oxygen atoms of the six-membered rings (6MRs) [13,14]. However, Kwak *et al.* identified two types of Cu ions at distinct cationic positions in Cu-SSZ-13 zeolite when Cu/Al is higher than 0.2 via H_2_-TPR and fourier transform infrared (FTIR) measurements [15]. Afterwards, researchers depicted the location and coordination of the two types of Cu ions (Fig. 4a) [16,17]. The relatively stable Cu^2+^ ions, which compensate paired negative charge (labelled as Cu^2+^-2Al), are located in the windows of 6MRs. After saturation of paired Al, [CuOH]^+^ ions appear subsequently and populate single framework Al sites next to 8MRs (labelled as [Cu(OH)]^+^-Al). Consequently, the amount of active copper species can be adjusted by tuning the Al contents and distribution. For example, with the increase of Al content (from Si-rich zeolites to Al-rich zeolites), the capacity for Cu^2+^-2Al species increases due to the presence of large amounts of paired Al, and the [Cu(OH)]^+^-Al species appear only at high Cu/Al [17]. The Al distribution at a fixed Si/Al ratio can be adjusted by using different structure-directing agents (SDAs) to synthesize the SSZ-13 substrate. Iorio *et al.* found that the density of paired Al increased linearly with the Na^+^ content incorporated into the crystallized solid when using TMAda^+^ and Na^+^ as organic and inorganic SDA [18,19]. Zhang *et al.* also increased the amounts of close Al sites in SSZ-13 by adjusting the crystallization pathways using DMCHA^+^ as the organic template, generating more Cu^2+^-2Al species than found in Cu-SSZ-13 synthesized using TMAda^+^ as the organic template [20].

The atmospheric environment can also influence the Cu state and coordination, and is closely related to the low-temperature catalytic activity of active copper species in Cu-SSZ-13 zeolite. Generally, the copper species are in the hydrated state in the ambient air, represented as [Cu(OH)(H_2_O)_5_]^+^ and [Cu(H_2_O)_6_]^2+^ [17,21]. The coordination between Cu ions and the zeolite structure can be weakened by the solvation effect of H_2_O. When increasing the temperature to ∼250°C, the hydrated copper species will release bound water and become bound at the ion-exchange sites in the form of [Cu(OH)]^+^-Al and Cu^2+^-2Al [21]. Similarly, the copper species are solvated by ammonia much more strongly than by H_2_O in the presence of NH_3_. The formed Cu-NH_3_ complex is thought to be the active site in the low-temperature NH_3_-SCR reaction [12,22–24]. More importantly, the active sites are movable within a limited region (∼9 Å) due to the weak bonding between Cu ions and the zeolite structure due to NH_3_ solvation (Fig. 4b), which further increases the effective collision possibility between active sites and reactants in a local area. Meanwhile, concomitant with the solvation of copper species by NH_3_, the reduction of Cu^2+^ ions takes place, resulting in NH_3_-solvated Cu^+^ ions [17,23]. Compared to Cu^2+^-2Al, [Cu(OH)]^+^-Al is more readily reducible by NH_3_ to form Cu(NH_3_)_n_^+^ and H_2_O [17]. The mobile NH_3_-solvated Cu^+^ species, which play a pivotal role in the NH_3_-SCR reaction, can also be generated in the actual NH_3_-SCR atmosphere. Researchers have also found the ‘auto-reduction’ of Cu^2+^ ions in a vacuum, inert atmosphere and even O_2_ atmosphere at high temperatures [21,25].

### Standard SCR reaction mechanism

**Figure 5.**
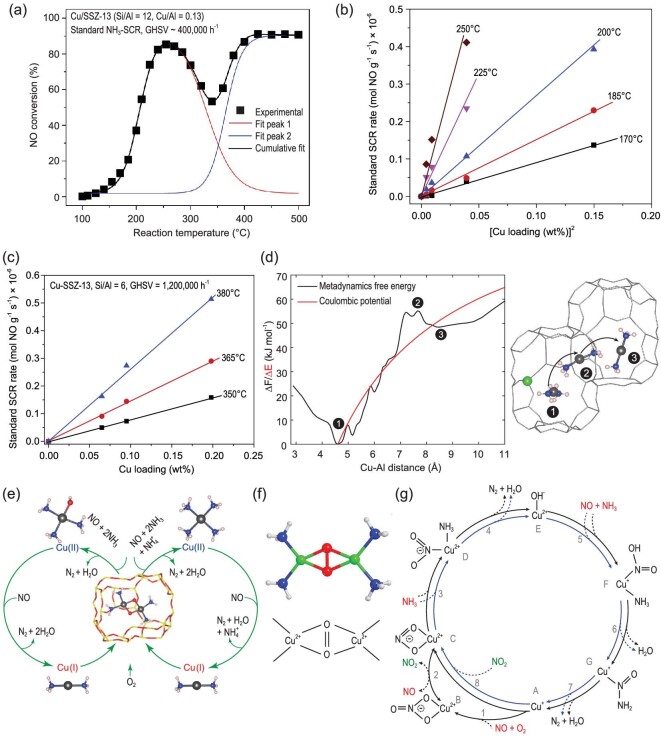
(a) NO conversion as a function of temperature over Cu-SSZ-13. Adapted with permission from ref. [22]. SCR rate over Cu-SSZ-13 as a function of (b) (Cu loading)^2^ at low temperatures and (c) (Cu loading) at high temperatures. Adapted with permission from ref. [27]. (d) Free energy and diffusion of Cu^I^(NH_3_)_2_ at different sites. Adapted with permission from ref. [12]. (e) Proposed low-temperature standard SCR catalytic cycle. Adapted with permission from ref. [12]. (f) Side-on μ-η^2^, η^2^-peroxo diamino dicopper (II) complex [Cu_2_(NH_3_)_4_O_2_]^2+^ species. Adapted with permission from ref. [31]. (g) Possible high-temperature SCR catalytic cycle. Adapted with permission from ref. [34].

Great efforts have been devoted to uncovering the standard SCR (SSCR) reaction cycle of Cu-SSZ-13, including the reduction (Cu^2+^→Cu^+^) and oxidation (Cu^+^→Cu^2+^) half-cycles. A seagull NO*_x_* profile (Fig. 5a) with dual-maxima NO*_x_* conversion was observed during the SSCR reaction over Cu-SSZ-13 catalysts with low Cu loading [22,26]. Figure 5b and c depict the linear relationship of the SSCR rate as a function of (Cu loading)^2^ and (Cu loading) at low and high temperatures, respectively [27]. Both of these indicate two distinct reaction regimes that require participation of paired Cu ions and isolated Cu ions at low and high temperatures, respectively.

A representative SSCR reaction cycle over Cu-SSZ-13 at low temperatures is shown in Fig. 5e. In the reduction half-cycle of SCR, Cu species which act as both redox and Lewis acid sites are readily reduced under the coexistence of NH_3_ and NO at low temperatures (below 250°C), leading to the formation of NH_3_-solvated Cu^+^ species, as discussed above [23,28,29]. In the oxidation half-cycle of SCR, binuclear Cu ions, which are achieved via the migration of Cu(NH_3_)_2_^+^, are essential for O_2_ activation based on the linear relationship between the standard SCR rate and (Cu loading)^2^ [22]. The electrostatic tethering between Cu(NH_3_)_2_^+^ and the zeolite structure becomes weak after NH_3_ coordination, resulting in dynamic multinuclear active sites in a limited volume (Fig. 5d). Paolucci *et al.* calculated that the diffusion radius of Cu(NH_3_)_2_^+^ is ∼9 Å, meaning that Cu(NH_3_)_2_^+^ can travel through 8MR CHA windows to form binuclear Cu ions [12]. This mechanism is a breakthrough in catalytic chemistry because it is outside the scope of traditional heterogeneous and homogeneous reactions, behaving more like a locally homogeneous reaction (Fig. 5e). The density functional theory (DFT) calculation result reveals that O_2_ activation (that is, formation of Cu(NH_3_)_2_-O_2_-Cu(NH_3_)_2_) is the rate-determining step for the NH_3_-SCR reaction over Cu-SSZ-13 [12,22,30]. Recently, Negri *et al.* identified the formation of a side-on μ-η^2^, η^2^-peroxo diamino dicopper (II) complex [Cu_2_(NH_3_)_4_O_2_]^2+^ (Fig. 5f) during the reaction of linear Cu(NH_3_)_2_^+^ with O_2_ via wavelet transform analysis of the extended X-ray absorption fine structure (EXAFS) data with other auxiliary measurements [31]. Four electrons are required to complete the O_2_ activation and dissociation. Therefore, NO was proposed to act as another electron provider in addition to two [Cu(NH_3_)]^+^, which can only deliver two electrons [22]. Using DFT calculations, Chen *et al.* investigated the reactivity of [Cu_2_(NH_3_)_4_O_2_]^2+^ with NO by proposing two cycles, in which NO is adsorbed on Cu(II) sites to form NO^+^ or oxygen to form NO_2_^−^. In their calculations, Brønsted acid sites play a pivotal role in the decomposition of HONO and H_2_NNO intermediates formed on Cu sites, and the catalytic cycle is probably controlled by the orientation of HONO and H_2_NNO from Cu sites to Brønsted acid sites [32]. By combining X-ray absorption spectroscopy (XAS) and ultraviolet-visible-near-infrared (UV-Vis-NIR) spectroscopies, Negri *et al.* found that NO + NH_3_ and NO can react with the [Cu_2_(NH_3_)_4_O_2_]^2+^ complexes, and an excess of the reactants leads to complete reduction of [Cu_2_(NH_3_)_4_O_2_]^2+^ to linear [Cu(NH_3_)]^+^ accompanied by the formation of N_2_, indicating the reaction of [Cu_2_(NH_3_)_4_O_2_]^2+^ with NO [31].

Turning to the reaction mechanism at elevated temperatures (>300°C), isolated Cu ions anchor on the ion-exchange sites and become fixed in the zeolite framework due to the decomposition of Cu(NH_3_)n species, according to NH_3_-TPD results [33]. The SSCR reaction activation energy at high temperatures (∼140 kJ/mol) is significantly higher than that at low temperatures (∼70 kJ/mol), indicating the presence of a different reaction pathway [27]. Janssens *et al.* proposed a reaction mechanism for the SSCR reaction based on isolated Cu^2+^/Cu^+^ ions [34], which is more like the reaction cycle at high temperatures (Fig. 5g). The Cu^2+^ ions are reduced by NO + NH_3_ and re-oxidized by NO + O_2_ or NO_2_. They indicated that the oxidation of an NO molecule by O_2_ to a form bidentate nitrate ligand is rate-determining for the SSCR.

Generally, most studies discuss the SSCR reaction cycle over the fresh Cu-SSZ-13 with low Cu loading, which simplifies the catalytic system with only a single Cu site. However, in actual application, the Cu loading of Cu-SSZ-13 is usually higher than the theoretical one. The NO reduction at high temperatures would decrease with the increase of Cu loading due to the occurrence of non-selective NH_3_ oxidation by oxygen at high temperatures, which leads to a lack of reductant. Moreover, some Cu species in the deep pores cannot gain access to the reactants. Therefore, controlling the amount of active Cu sites is important for actual application of Cu-SSZ-13. In another aspect, when Cu-SSZ-13 is aged, the behaviour is much more complicated due to the presence of various Cu species (such as CuO_x_). As a result, there have been few studies focusing on aged or high Cu-loaded Cu-SSZ-13. However, more studies should be conducted to understand the reaction process of aged or high Cu-loaded Cu-SSZ-13 zeolites, since these catalysts are closer to the actual situation in practice.

### FSCR reaction and NH_4_NO_3_ formation

**Figure 6.**
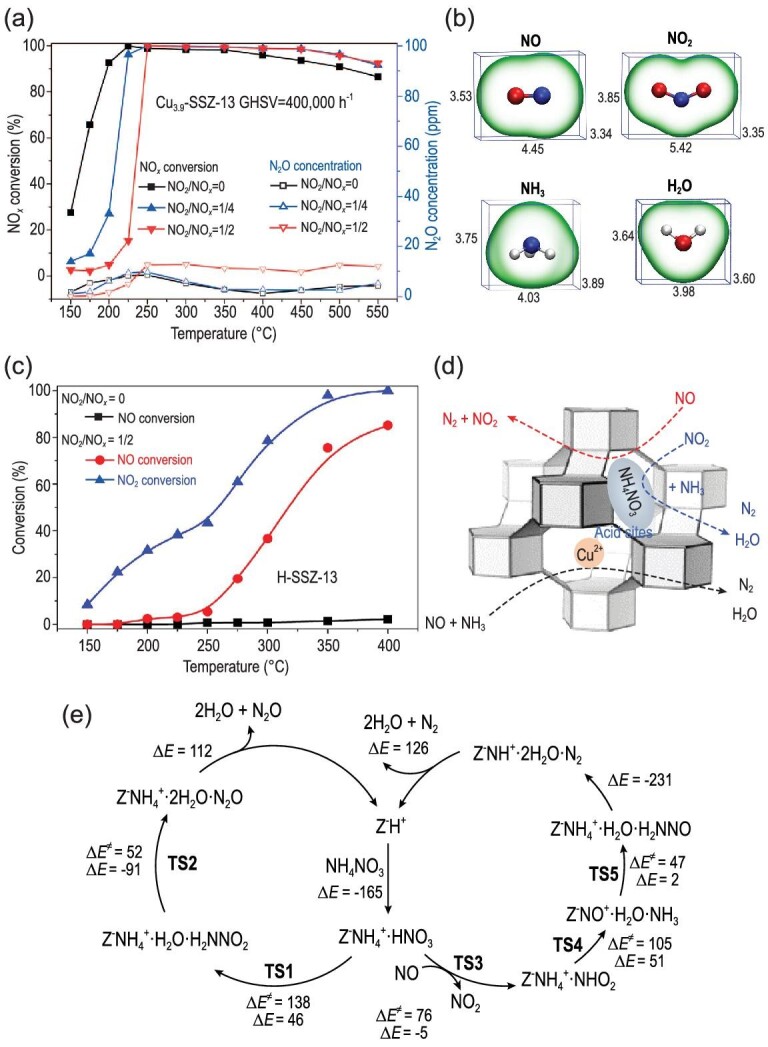
(a) NH_3_-SCR performance of Cu-SSZ-13 under different reaction conditions. Adapted with permission from ref. [36]. (b) Different reaction pathways of NO and NO_2_ over Cu-SSZ-13. Adapted with permission from ref. [37]. (c) NO, NO_2_ and NO*_x_* conversion on H-SSZ-13 under fast SCR conditions. Adapted with permission from ref. [37]. (d) Molecular size of NH_3_, NO and NO_2_. Adapted with permission from ref. [37]. (e) DFT-calculated reaction pathways for direct and NO-assisted NH_4_NO_3_ decomposition. Adapted with permission from ref. [39].

The FSCR reaction (reaction (2)) also plays an important role in NO*_x_* reduction due to the presence of ∼10% NO_2_ in actual diesel exhaust, as well as the partial oxidation of NO to NO_2_ in the DOC section. Normally, NO_2_ addition to the feed is an effective way of achieving high deNO*_x_* efficiency for NH_3_-SCR catalysts. Differently to most NH_3_-SCR catalysts, however, Cu-SSZ-13 catalysts only showed a slight improvement in NO*_x_* reduction under FSCR conditions, as reported by Kwak *et al.* [35]. Our group even found an inhibition effect on NO*_x_* conversion over our *in situ* synthesized Al-rich Cu-SSZ-13 catalyst due to the formation of ammonium nitrate (Fig. 6a) [36,37]. Therefore, the small-pore zeolites scarcely rely on NO oxidation to achieve the FSCR reaction, unlike other SCR catalysts. The formation of surface NH_4_NO_3_ from the adsorbed NO_2_ and NH_3_ is a common reaction in the FSCR according to reaction (7), and NH_4_NO_3_ decomposition primarily takes place in two ways as shown in reaction (8) and reaction (9):

(7) \begin{equation*}2{\rm{N}}{{\rm{O}}_2} + 2{\rm{N}}{{\rm{H}}_3} \to {{\rm{N}}_2} + {\rm{N}}{{\rm{H}}_4}{\rm{N}}{{\rm{O}}_3} + {{\rm{H}}_2}{\rm{O}}\end{equation*}

(8) \begin{equation*}{\rm{N}}{{\rm{H}}_4}{\rm{N}}{{\rm{O}}_3} \to {{\rm{N}}_2}{\rm{O}} + 2{{\rm{H}}_2}{\rm{O}}\end{equation*}

(9) \begin{equation*}{\rm{N}}{{\rm{H}}_4}{\rm{N}}{{\rm{O}}_3} + {\rm{NO}} \to {\rm{N}}{{\rm{O}}_2} + {{\rm{N}}_2} + 2{{\rm{H}}_2}{\rm{O}}\end{equation*}

The comparable geometric dimensions of the small pores (∼3.8 Å) and reactant molecules (Fig. 6b) are mainly responsible for the strong inhibition of NO conversion activity, since a slight amount of NH_4_NO_3_ accumulation can limit the diffusion of reactants as well as [Cu(NH_3_)_2_]^+^ species. Moreover, the NH_4_NO_3_ formed in small-pore zeolites is more stable than that in large-pore zeolites [38]. Our group investigated the effect of NO_2_ on the NH_3_-SCR reaction over Cu-SSZ-13 with varying Cu loadings [37]. We found that increased Cu loadings contributed to the SSCR reaction, while inhibiting the FSCR reaction. At low temperatures, the NO conversion activity was totally suppressed because of the formation of NH_4_NO_3_, while NO_2_ could react with NH_3_ at acid sites to contribute to NO*_x_* conversion (Fig. 6d). Further, it was observed that the deposited NH_4_NO_3_ reacted with NO over H-SSZ-13 at high temperatures (Fig. 6c), accompanied by the direct decomposition of NH_4_NO_3_ to N_2_O (reaction (8)). However, the N_2_O production is less than 25 ppm. As shown in Fig. 6e, the DFT-calculated reaction pathways demonstrated that the energy barrier (105 kJ/mol) for NO-assisted NH_4_NO_3_ decomposition (reaction (9)) is much lower compared to that (138 kJ/mol) of direct NH_4_NO_3_ decomposition (reaction (8)) [39]. The combination of reaction (9) and reaction (7) yields the FSCR reaction (2), indicating that the FSCR reaction occurs only on Brønsted acid sites, which is also testified to by computational methods [39,40]. To avoid this inhibition phenomenon, we conducted hydrothermal aging to decrease the density of Brønsted acid sites to alleviate NH_4_NO_3_ accumulation and cause mesopore formation to favour gas diffusion [41]. As expected, the inhibiting effect of NO_2_ on NO*_x_* conversion was weakened for the hydrothermally aged Cu-SSZ-13 catalysts. In another aspect, what should be noted is that NH_4_NO_3_ accumulated only under steady-state conditions. Bendrich *et al.* found that almost no NH_4_NO_3_ is predicted to form during temperature oscillation and NO/NO_2_ ratio oscillation [42,43]. The decomposition temperature of NH_4_NO_3_ formed on Cu-SSZ-13 is about ∼225°C, which is easily exceeded during the driving cycle [37]. As for oscillating NO/NO_2_ ratios, NH_4_NO_3_ can act as an NO_2_ buffer that stores NO_2_ under NO_2_-excess conditions and decomposes under NO_2_-deficient conditions.

There is a common consensus that NH_4_NO_3_ forms on Cu-SSZ-13 and inhibits NO*_x_* conversion under FSCR conditions at steady-state and low temperatures. An atomic-level picture of the FSCR reaction cycle is still lacking, especially regarding the Cu sites. Research studies have only found Cu(II) species, due to the strong oxidizing ability of NO_2_, and Cu(NH_3_)n complexes have scarcely been identified under FSCR conditions, making it difficult to investigate the reduction and oxidation half-cycle of the SCR reaction [12,44]. Moreover, the NH_4_NO_3_ formation and high reactivity of the SSZ-13 substrate limit the investigation of Cu active sites. The reaction cycle containing NO_2_ shown in Fig. 5g is a rational candidate due to the presence of Cu(II) species coordinated strongly to zeolite and the absence of the Cu(NH_3_)_n_ complex. However, the evidence is not conclusive, and more studies are required in future.

### Hydrothermal stability

For the aftertreatment system of diesel engines, excellent hydrothermal stability (HTS) is an indispensable property due to the high-temperature environment (above 650°C) resulting from regeneration of the upstream DPF. Cu-SSZ-13 small-pore zeolites were reported to have superior HTS to that of medium- and large-pore zeolites as well as most of the oxide catalysts [11]. Researchers have devoted much effort to unravelling the deactivation mechanism of hydrothermal aging (HTA).

**Figure 7.**
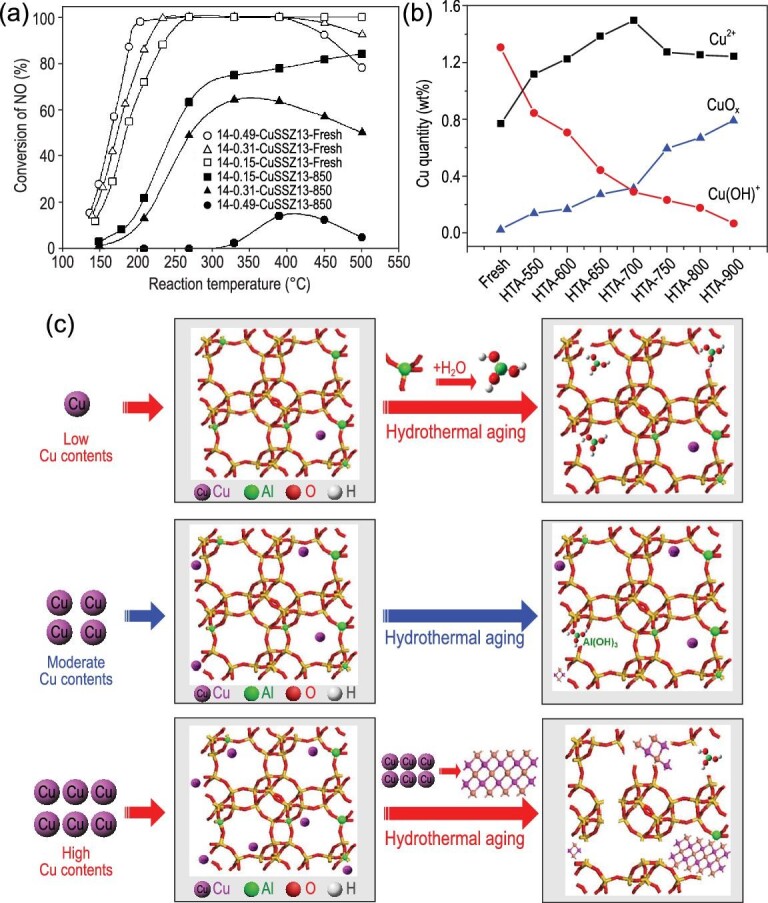
(a) Standard NH_3_-SCR performance of fresh and HTA Cu-SSZ-13 with different Cu/Al ratios. Adapted with permission from ref. [46]. (b) Estimation of Cu^2+^-2Al, [Cu(OH)]^+^-Al and CuO*_x_* in fresh and HTA samples. Adapted with permission from ref. [48]. (c) The deactivation mechanism of HTA of Cu-SSZ-13 with different Cu contents. Adapted with permission from ref. [51].

Hydrolysis of framework Al and accumulation of CuO*_x_* from Cu^2+^ active sites are the two key reasons for HTA deactivation. The narrow small pores (∼3.8 Å) block the diffusion of hydrolysed Al(OH)_3_, with large kinetic diameter of ∼5.03 Å, leading to its reattachment back into the structural defects caused by dealumination when the zeolite cools down [45]. This is the principal reason that small-pore zeolites have higher skeleton stability than medium- and large-pore zeolites during HTA. Regarding the effect of copper species, researchers found that HTS decreased with increasing Cu/Al ratio (Fig. 7a) [45–47]. As mentioned in ‘Copper species’ section, Cu^2+^-2Al species with higher stability primarily exist in Cu-SSZ-13 catalysts with low Cu loading. Increasing Cu loading leads to formation of [Cu(OH)]^+^-Al species, which easily accumulate to form CuO*_x_*. This was also proved by Gao and co-workers using electron paramagnetic resonance (EPR) as the primary measurement [48,49]. They found that mild HTA first induced some conversion of [Cu(OH)]^+^-Al to Cu^2+^-2Al species, resulting in paired Cu^2+^ located in a D6R prism with a distance of ∼3.90 Å [49]. Further increasing the HTA temperature, [Cu(OH)]^+^-Al species gradually accumulated to form CuO*_x_* clusters (Fig. 7b) [48].

The Si/Al ratio can also influence the HTS of Cu-SSZ-13 by affecting the copper distribution. Al-rich zeolites favour the presence of Cu^2+^-2Al species, thus leading to higher HTS compared to Si-rich zeolites [46,50]. Nevertheless, the Al-rich zeolites still face the problem of dealumination, which will cause the loss of Brønsted acid sites during HTA, due to the large amount of vulnerable framework Al. We investigated the HTS of Al-rich Cu-SSZ-13 zeolite and found that Cu^2+^ ions inhibit dealumination of the SSZ-13 zeolite structure, while excessive quantities of them easily accumulate to form CuO*_x_* clusters, leading to collapse of long-range order (Fig. 7c) [51]. Ye *et al.* also reported that preservation of active Cu^2+^ sites is more important than preserving Brønsted acid sites [52]. This is understandable, because Brønsted acid sites only serve as an NH_3_ reservoir or probably as active sites for the decomposition of HONO and H_2_NNO intermediates, as discussed in ‘Standard SCR reaction mechanism’ section, while the Cu^2+^ species can act as both redox and acid sites and can complete the NH_3_-SCR reaction cycle on their own. Besides CuO*_x_*, CuAlO*_x_* species were also detected by researchers [53–55]. Ma *et al.* found that CuAlO*_x_* originating from the combination of Cu with extra-framework Al is inert, while CuO_x_ species still have catalytic oxidation ability [55]. Schmidt *et al.* found evidence that Cu-SSZ-13 only shows Cu and Al clustering separately, while Cu-ZSM-5 contains large amounts of copper aluminate species after HTA, by using atom probe tomography technology, which can show the 3D distributions of component elements [54].

In summary, CuO*_x_* formation and dealumination are two reasons for HTA deactivation, and interact during HTA. CuO_x_ accumulation can induce zeolite structure collapse, and extra-framework Al can coordinate with Cu to form CuAlO*_x_* species that are inert. As both redox and acid sites, Cu species are of vital importance and should be carefully tuned and protected. As an alternative, utilizing an Al-rich zeolite support is a simple and effective way to increase the HTS of Cu-SSZ-13 since the capacity for stable Cu^2+^-2Al species is large due to the presence of many paired Al atoms in Al-rich zeolites.

### Sulphur poisoning and desulphurization

**Figure 8.**
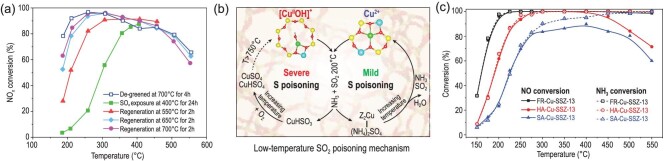
(a) Effect of SO*_x_* exposure and subsequent thermal deSO*_x_*. Adapted with permission from ref. [56]. (b) Proposed formation of sulphates during adsorption of SO_2_ on [Cu(OH)]^+^-Al and Cu^2+^-2Al sites. Adapted with permission from ref. [61]. (c) NH_3_-SCR performance of fresh Cu-SSZ-13 (FR-Cu-SSZ-13), hydrothermally aged Cu-SSZ-13 (HA-Cu-SSZ-13) and sulphur-aged Cu-SSZ-13 (SA-Cu-SSZ-13) at high temperatures (750°C). Adapted with permission from ref. [64].

Sulphur poisoning is an inevitable challenge due to the residual sulphur in diesel fuel. Figure 8a shows the SSCR performance of fresh, sulphated and subsequently desulphated Cu-SSZ-13 catalysts [56]. The low-temperature performance was significantly inhibited on the sulphated samples compared to fresh ones, while the high-temperature performance was scarcely influenced. Thermal treatment can partially recover the deNO*_x_* efficiency of Cu-SSZ-13 and the low-temperature activity increased with progressively higher regeneration temperatures, indicating that various S-containing species, including reversible and irreversible ones, existed on the sulphated Cu-SSZ-13 catalysts.

Low-temperature (200°C) SO_2_ poisoning of Cu-SSZ-13 generally leads to the formation of H_2_SO_4_, CuSO_4_ and Al_2_(SO_4_)_3_ species, with different thermal stabilities [57]. The decomposition temperatures are ∼500°C, ∼630°C and ∼800°C for H_2_SO_4_, CuSO_4_ and Al_2_(SO_4_)_3_, respectively [57]. H_2_SO_4_ easily combines with NH_3_ to promote the accumulation of ammonium-sulphur species under SCR conditions [57–60]. Therefore, SO_2_ treatment under SCR conditions (NH_3_ + NO + O_2_ + H_2_O) induced more severe deactivation compared to that in the presence of O_2_ + H_2_O only [58]. Jangjou *et al.* found that ammonium sulphate formed on Cu^2+^-2Al sites and could be generally regarded as a reversible deactivation due to its facile regeneration by heat treatment [61]. However, SO_2_ adsorbed on [Cu(OH)]^+^-Al sites leads to copper bisulphite species formation, which are further oxidized to form copper bisulphate with increasing temperature (Fig. 8b) [61]. The accumulation of ammonium sulphate and copper bisulphate limits the formation and mobility of [Cu(NH_3_)_2_]^+^ ions that serve as dynamic active sites, thus resulting in a marked decrease in deNO*_x_* efficiency [62,63]. The copper-sulphur species need a higher temperature (>550°C) to decompose and are thus thought to result in irreversible deactivation. Al_2_(SO_4_)_3_ is difficult to decompose and regarded as an irreversible deactivation species due to the destruction of framework Al. When the SO_2_-poisoning temperature increased to 400°C, more copper-sulphur species formed due to the instability of ammonium sulphate. However, 750°C sulphur aging with H_2_O resulted in permanent deactivation of Cu-SSZ-13 without accumulation of reversible species, since SO_2_ increased the acidity of the hydrothermal atmosphere and accelerated the destruction of the zeolite structure as well as the loss of acid sites and active Cu^2+^ species (Fig. 7c) [64]. Compared to SO_2_, SO_3_ poisoning is more severe due to the primary formation of copper bisulphate species, which only decompose at elevated temperatures (>550°C) [59].

Turning to the solution to SO_2_ poisoning, heat treatment at elevated temperature (∼500°C), an easily accessible temperature in actual diesel exhaust, is generally an effective way to regenerate the deNO*_x_* efficiency of Cu-SSZ-13, since most of the total deactivation is reversible. Moreover, it should be noted that some reductants, such as NH_3_ and C_3_H_6_, can achieve the removal of sulphur species by chemical reaction without heat treatment at elevated temperatures [56,65]. Besides, Wei *et al.* found that mild HTA increased the SO_2_ tolerance of Cu-SSZ-13 since some sulphur-sensitive [Cu(OH)]^+^-Al species transformed to stable Cu^2+^-2Al species during mild HTA [66]. Yu *et al.* found that some metal oxides, which can be mixed with Cu-SSZ-13 zeolite to form hybrid catalysts, can serve as a sacrificial component to prevent Cu^2+^ site poisoning. However, the improvement in SO_2_ tolerance is still limited [67].

### Effects of PO_4_^3−^, HCs and metal co-cations

The exhaust from diesel engines includes various contaminants derived from the engine, urea (NH_3_ source), volatile engine oils, volatile precious metals and fuel additives; therefore, besides hydrothermal aging and sulphur poisoning there are also other toxic species that need to be considered in diesel exhaust, such as PO_4_^3−^, HCs and alkali metals, although their quantities are small. Phosphorus mainly comes from fuels (biofuels) and some lubricating oils as well as some fuel additives. There have been several studies mainly focused on phosphorus poisoning of Cu-SSZ-13. Phosphorus poisoning is generally simulated by incipient wetness impregnation using (NH_4_)_2_HPO_4_ solutions as precursors. After PO_4_^3−^ loading, Cu-P species and Al-P species are formed and have been identified by many researchers [68–71]. The formation of Cu-P species induces a decrease in the NO and NH_3_ oxidation of Cu-SSZ-13, which further results in a reduction in low-temperature SCR activity due to loss of active Cu^2+^ sites, but improvement in high-temperature SCR activity due to the suppression of NH_3_ non-selective oxidation [68,71,72]. In detail, Wang *et al.* found that [Cu(OH)]^+^-Al interacts more easily with phosphorus and coordinates with only one P atom (PO_3_^−^ or PO_4_^3−^), while the poisoning of Cu^2+^-2Al involves two P atoms (i.e. P_2_O_5_). The formation of Al-P species primarily influences the HTS of Cu-SSZ-13. P loading can lead to part of the phosphorus inserting into the zeolite framework to form a localized AlPO_4_ phase, which is severe after HTA [72,73]. This indicates that P poisoning accelerates the collapse of the structure of Cu-SSZ-13 zeolite during HTA. However, Zhao *et al.* found, importantly, that an appropriate content of phosphate ions can prevent the structure collapse due to the formation of a silicoaluminophosphate interface [71]. To create conditions approaching the practical situation, Xie *et al.* simulated the vapour-phase phosphorus poisoning (using diluted H_3_PO_4_ solution) of Cu-SSZ-13 during NH_3_-SCR operating conditions [70]. Ammonium phosphate is easily formed at low temperatures but decomposes at elevated temperatures. Unfortunately, the decomposition of ammonium phosphate still deposits P in the catalysts. At high temperatures, metaphosphate (PO_3_^−^) was the dominant deposited compound compared to phosphorus oxide (P_2_O_5_) and phosphate (PO_4_^3−^) during PO_4_^3−^ poisoning under SCR conditions. In summary, PO_4_^3−^ poisoning results in accumulative and permanent deactivation, although a small amount of phosphorus probably benefits the hydrothermal stability.

The effect of co-cations should also be considered since the exhaust contains some metal impurities derived from various additives, and some metals are residual species or are purposefully added in the synthetic process to improve the catalytic activity of Cu-SSZ-13. The influence of Na on Cu-SSZ-13 has been extensively studied by researchers, since Na is not only a poison but also a residual species in most synthetic methods. We first investigated the effect of Na^+^ on one-pot synthesized Cu-SSZ-13 and basically found that the presence of Na^+^ decreased the catalytic activity and hydrothermal stability [74]. However, Gao *et al.* found that Na^+^, as well as Li^+^ and K^+^, promoted the low-temperature SCR rate and HTS of Cu-SSZ-13 [75,76]. Zhao also found that an appropriate Na^+^ content could increase both the low-temperature activity and hydrothermal stability of their organotemplate-free synthesized Cu-SSZ-13 [77]. The primary reason for the discrepancy is varying Cu contents in the Cu-SSZ-13 catalysts. Na^+^, located at ion-exchange sites, weakens interactions between Cu^2+^ and the zeolite framework and promotes some Cu^2+^ accumulation during HTA, which is more pronounced in the one-pot synthesized Cu-SSZ-13 with high Cu content due to the low Si/Al ratio (∼4.2). However, if the Cu content is suitable, the weakened interaction with the zeolite framework makes Cu^2+^ more reducible, and a moderate amount of Na^+^ can prevent zeolite dealumination and stabilize the framework during HTA. Analogously, among rare earth co-cations, moderate amounts of Ce and Y ions, which locate at ion-exchange sites, were found to improve the HTS of Al-rich Cu-SSZ-13 [78–81]. Other metal co-cations (such as Cs^+^, Ca^2+^ Mg^2+^ etc.) generally showed a poisoning effect on Cu-SSZ-13 since they destabilized isolated Cu ions via site competition [76,82]. In summary, some co-cations have a positive effect on Cu-SSZ-13, but controlling the content of Cu ions and co-cations is the key factor.

Hydrocarbon species are primarily formed due to insufficient combustion of fuel during cold-start oxidation or when the upstream DOC is aged. Basically, heavy HCs such as C_10_H_22_ and C_12_H_26_ have no effect on the deNO*_x_* efficiency of Cu-SSZ-13, since the small channel (∼3.8 Å) prevents the long-chain HC molecules from entering into the zeolite [83,84]. As for light HCs, Cu-SSZ-13 shows a slight decrease in NO conversion in the presence of C_3_H_6_ in the medium temperature range (200–400°C). In this temperature range, C_3_H_6_ is partially oxidized and forms some carbonaceous deposits, resulting in a decrease in NO conversion [83,85]. When the temperature is above 400°C, the deposited carbon burns off and C_3_H_6_-SCR can also occur, so that the NO_x_ conversion is only slightly changed [85–87]. When the temperature is below 200°C, there is also no adverse impact of C_3_H_6_ since the energy is insufficient to propel the C_3_H_6_ molecule (with a kinetic diameter of ∼4.5 Å) into the pores [85].

### Synthetic methodology

The availability of an efficient, economical and environmentally friendly synthetic method is vitally important for the practical application of Cu-SSZ-13. In the synthesis process, the temperature, seeds and template all influence the space-time yields (STY) of CHA zeolite. Generally, Cu-SSZ-13 catalysts are prepared by an ion-exchange method, with the SSZ-13 substrate synthesized using N,N,N-trimethyl-1-adamantammonium hydroxide (TMAdaOH) as a template [88]. In consideration of the low STY (160°C for 90–120 h), high price of TMAdaOH, and complex ion-exchange process, many new synthetic methods were developed by researchers. Han *et al.* used coal gangue as the Al source and shortened the nucleation time of SSZ-13 to 7 h in the presence of TMAdaOH, but 36 h was still needed to prepare SSZ-13 with high crystallinity [89]. Wang *et al.* used a solvent-free method to successfully synthesize SSZ-13 substrate by using economical N,N,N-dimethylethylcyclohexylammonium bromide (DMCHABr) as an organic template to achieve 95.7% of efficiency of silica source usage. Interestingly, the Cu^2+^ ion-exchanged SSZ-13 synthesized using DMCHABr as a template showed higher HTS than Cu-SSZ-13 synthesized by the traditional method using TMAdaOH as a template, since more spatially close Al sites (favouring Cu^2+^-2Z as elaborated in ‘Hydrothermal stability’ section) exist in the former Cu-SSZ-13 [20,90]. Furthermore, seed-assisted rapid synthesis of SSZ-13 through interzeolite transformation from FAU zeolite was achieved in the absence of solvent, which shortened the crystallization time to 1 day [91]. Simultaneously, the solvent-free conditions benefit the stability of the TMAdaOH template at high temperatures (>200°C), so that Bian *et al.* shortened the synthetic time of SSZ-13 to only 1.5 h (extremely high STY) at 240°C. Besides DMCHABr, Chen *et al.* used economical choline chloride (CC) as a template to synthesize the SSZ-13 substrate [92]. Furthermore, they optimized this method with the assistance of fluoride and seeds, resulting in crystallinity similar to that of a sample synthesized using TMAdaOH [93]. Moreover, through the utilization of CHA seeds, an organotemplate-free synthesis of SSZ-13 was developed by Zhao *et al.* [77]. After Cu^2+^ ion-exchange, this Cu-SSZ-13 sample showed excellent catalytic activity, with NO*_x_* conversion higher than 90% in the temperature range 200–500°C, but at relatively low GHSV (80 000 h^−1^).

**Figure 9.**
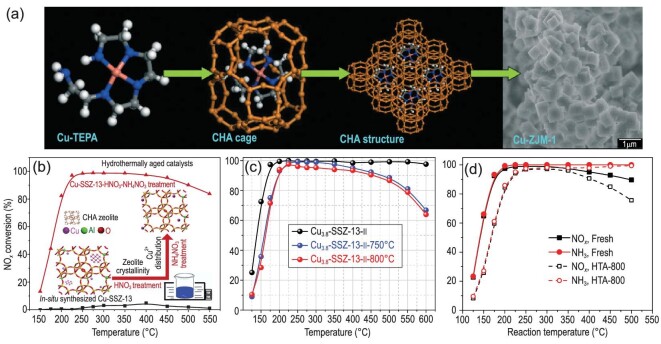
(a) Mechanism of preparation of Cu-TEPA templated Cu-SSZ-13 zeolites. Adapted with permission from ref. [94]. (b) HNO_3_-NH_4_NO_3_ aftertreatment of *in situ* synthesized Cu-SSZ-13. (c) NH_3_-SCR performance of aftertreated Cu-SSZ-13 before and after hydrothermal aging at 750 and 800°C, GHSV = 200 000 h^−1^. Adapted with permission from ref. [51]. (d) NH_3_-SCR performance of commercial fresh and HTA-800 Cu/SSZ-13 samples (Si/Al = 12, Cu loading = 2.1%), GHSV = 200 000 h^−1^. Adapted with permission from ref. [48].

The product synthesized by all the above methods is the SSZ-13 substrate, which subsequently needs multiple steps to obtain Cu-SSZ-13 catalysts, at least including calcination, NH_4_^+^ ion-exchange, Cu^2+^ ion-exchange and calcination again. Therefore, a direct synthesis method for Cu-SSZ-13 catalyst is desired. Xiao and co-workers first directly synthesized Cu-SSZ-13 by using Cu-tetraethylenepentamine (Cu-TEPA) as a template to introduce the copper species *in situ* (Fig. 9a) [94]. Nevertheless, using Cu-TEPA as the sole template easily led to the formation of accumulation of CuO*_x_* clusters, which is detrimental to HTS and high-temperature activity (Fig. 9b, black line). Therefore, a series of aftertreatments were developed by our group to optimize the structure and copper species of *in situ* synthesized Cu-SSZ-13 [51,74,86]. We found that synergetic treatment by HNO_3_ and NH_4_NO_3_, which can accurately tune the framework crystallinity and copper state and content, markedly increased the SCR activity and hydrothermal stability (Fig. 9b, red line). Since Cu-SSZ-13 was first reported to have excellent NH_3_-SCR activity and hydrothermal stability in 2010, researchers have studied the physiochemical properties of Cu-SSZ-13 in detail and optimized the Si/Al ratio to ∼12 from 17.5 [48,95], and we believe that the Si/Al of Cu-SSZ-13 can be lowered further. It should be noted that the *in situ* synthesized Cu-SSZ-13 (Fig. 9c) showed activity superior to commercial Cu-SSZ-13 (Fig. 9d, Si/Al∼12), especially at high temperatures. This is because the *in situ* synthesized Cu-SSZ-13 is an Al-rich zeolite, which accommodates large amounts of paired framework Al for stable coordination of Cu^2+^-2Al (see details in ‘Copper species’ section), thus leading to high deNO*_x_* efficiency and HTS.

## OTHER SMALL-PORE ZEOLITES APPLIED TO NH_3_-SCR

### Shape selectivity of small-pore structure in NH_3_-SCR reaction

Cu-SSZ-13 is a Cu-exchanged small-pore zeolite with the CHA crystal structure, which contains a channel opening of about 3.8 × 3.8 Å (8MR) and a large CHA cage. As stated above, this specific structure is closely related to the properties of Cu-SSZ-13 catalysts in the NH_3_-SCR reaction. For example, the dealumination process of small-pore zeolites during HTA is inhibited because the constricting dimensions of the small pores limits the detachment of aluminium hydroxide [45,96]. The small-pore structure restricts large hydrocarbon species from entering the pores of the catalysts during the SCR reactions, so that Cu-SSZ-13 possesses good hydrocarbon tolerance, especially towards long-chain HCs [45]. The small-pore Cu-CHA zeolite showed very slow N_2_O formation in the NH_3_-SCR reaction because the small-pore structure can stabilize NH_4_NO_3_ [38]. Moreover, the formation of dynamic binuclear Cu ions can be achieved in the large CHA cage. Therefore, the HTS, poisoning resistance, catalytic selectivity and activity, and reaction mechanism are closely related to the shape selectivity of the SSZ-13 structure.

Based on the shape selectivity of the Cu-SSZ-13 catalyst, here, we summarize the essential characteristics of Cu-based small-pore zeolites with excellent activity and hydrothermal stability: (i) The 8MR pore structure is extremely important to its HTS due to its inhibiting effect on dealumination. Moreover, NH_3_-SCR reactants with small volume go through the 8MR easily while some large poisons (such as long-chain HCs) are prevented from entering the zeolite. (ii) A suitable elemental composition offers plenty of ion-exchangeable sites and Brønsted sites as well as a stable skeleton. (iii) The large cage-type structure (CHA cage of SSZ-13) provides a reaction zone where a large molecule such as dynamic Cu(NH_3_)_2_^+^ can favourably combine to form di-nuclear active sites to complete the O_2_ activation. Based on the above principles, researchers have surveyed the zeolite family, and some typical small-pore zeolites with comparable deNO*_x_* efficiency or/and HTS to Cu-SSZ-13 were developed.

### Hydrothermally stable small-pore zeolites

**Figure 10.**
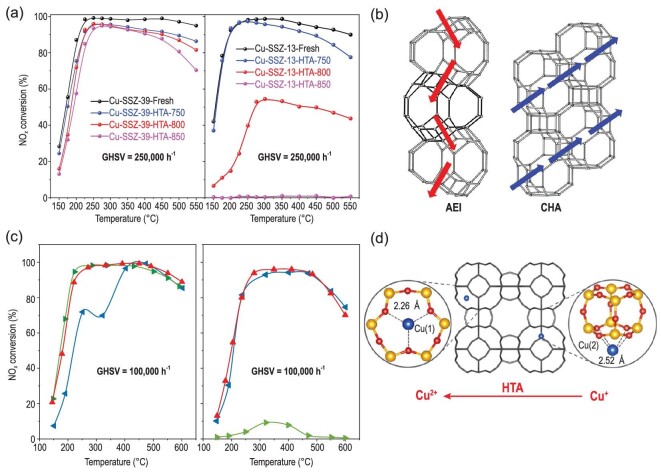
(a) Comparative NH_3_-SCR performance of Cu-SSZ-39 and Cu-SSZ-13 before and after hydrothermal aging. (b) Channel structures of AEI and CHA zeolites. Adapted with permission from ref. [98]. (c) NH_3_-SCR performance of Cu-LTA-16–0.48 (red), Cu-LTA-11–0.48 (green) and Cu-LTA-23–0.5 (blue) before (left) and after (right) hydrothermal aging at 900°C. Adapted with permission from ref. [104]. (d) Copper species at different locations and transformation between the two copper species during HTA. Adapted with permission from ref. [105].

Utilization of the Cu-SSZ-39 zeolite catalyst with AEI structure for the SCR of NO*_x_* was first reported by Moliner *et al.* [97]. The difference between the AEI and CHA structures is the connection mode of the D6Rs. The neighbouring D6Rs have mirror symmetry in AEI, while they are arranged in parallel in CHA, resulting in different channels and cavities. Recently, we compared the NH_3_-SCR activity and HTS of Al-rich Cu-SSZ-39 and Cu-SSZ-13 and found that Cu-SSZ-39 showed higher hydrothermal stability but lower deNO*_x_* efficiency (Fig. 10a), and both of them showed excellent N_2_ selectivity, with N_2_O production less than 10 ppm [98]. It was found that SSZ-39 contained more paired Al, which favoured the formation of stable Cu^2+^-2Al species, resulting in higher stability for active sites but lower deNO*_x_* efficiency compared to [Cu(OH)]^+^-Al. In another aspect, SSZ-39 has a more tortuous channel structure than SSZ-13 does (Fig. 10b). This structure can inhibit the detached Al(OH)_3_ from exiting the pores of the AEI zeolite framework, which would result in reincorporation of Al(OH)_3_ into the framework when the catalyst cools down. However, the tortuous channel is adverse to the mobility of active Cu(NH_3_)^+^ species, which further reduces the deNO*_x_* efficiency [98]. Furthermore, we investigated the SO_2_, alkali and alkaline earth metal resistance of Cu-SSZ-39 for NH_3_-SCR. Similar to Cu-SSZ-13, Cu-SSZ-39 also showed reversible (H_2_SO_4_) and irreversible (CuSO_4_) deactivation after SO_2_ poisoning. Regeneration at 600°C can recover most of the NH_3_-SCR activity, but decomposition of CuSO_4_ needs a higher temperature [99]. However, the catalytic activity and HTS were significantly decreased after alkali/alkaline earth poisoning due to the deterioration of the zeolite structure and CuO*_x_* formation from isolated Cu^2+^, which still should be optimized and improved [100]. To further increase the hydrothermal stability of Cu-SSZ-39, Sano and co-workers used tetraethylphosphonium (TEP) cations as a structure-directing agent to obtain P-modified Cu-SSZ-39 with excellent hydrothermal stability (hydrothermal treatment at 900°C for 4 h). However, fluoride, which is an environmental pollutant and harmful to human health, was required to accelerate mineralization, and NO conversion decreased with increasing P/Al ratio [101]. Martin *et al.* used N,N-dimethyl-3,5-dimethylpiperidinium and Cu-TEPA as dual OSDAs to directly synthesize Cu-SSZ-39, which showed good NH_3_-SCR performance, but HTS was not tested [102]. Moreover, given that SSZ-39 has traditionally been synthesized through interzeolite transformation from high-silica Y, of which the preparation is expensive and requires complex post-treatments, Xu *et al.* successfully synthesized SSZ-39 through interzeolite transformation from low-cost and widely used ZSM-5 and beta zeolites [103]. In summary, Cu-SSZ-39 zeolite exhibits strong potential as an NH_3_-SCR catalyst for actual application due to its optimal deNO*_x_* efficiency and outstanding hydrothermal stability.

Besides Cu-SSZ-39, high silica Cu-LTA is another highly stable zeolite for the NH_3_-SCR reaction. It was first reported by Hong *et al.* that fully copper-exchanged high-silica LTA zeolite possessed excellent hydrothermal stability during the NH_3_-SCR reaction. Even after hydrothermal aging at 900°C, the samples still showed good NO*_x_* conversion (Fig. 10c), under which conditions the Cu-SSZ-13 structure collapsed [104]. Furthermore, an increase in the low-temperature standard NH_3_-SCR activity was found on Cu-LTA with Si/Al of 23 and Cu/Al of 0.5 after hydrothermal aging. They ascribed this to the transformation of inactive Cu^+^ ions in the sod cage coordinated to four-rings to active Cu^2+^ ions in the single 6-rings (s6r) centre (Fig. 10d) [105]. In addition, Wang *et al.* found that HTA prompted the CuO*_x_* and Cu^2+^ to transform to Cu(OH)^+^ species on a Cu/LTA catalyst prepared by the incipient wetness impregnation (IWI) method, which is opposite to the behaviour of Cu-SSZ-13 [106]. Another aspect that is different from Cu-SSZ-13 is the promotion effect on NO*_x_* conversion of Cu-LTA under FSCR conditions [106,107]. Ryu *et al.* found that both Cu^2+^ and Cu(OH)^+^ in Cu-LTA are substantially centred on the single six-rings, where the reactant molecules should be less accessible than the eight-ring window sites, resulting in lower amounts of ammonia nitrate compared to that produced in Cu-SSZ-13 [107]. Regarding the effect of SO_2_, the Cu(OH)^+^ species in this catalyst were found to be more vulnerable to SO_2_ poisoning due to their weak interaction with the zeolite framework structure compared to those in Cu-SSZ-13. Nevertheless, the fresh deNO*_x_* efficiency of Cu-LTA can be totally recovered by regeneration at the elevated temperature of 750°C [108]. Although Cu-LTA zeolite showed extraordinary HTS, relatively optimized FSCR performance as well as good SO_2_ tolerance, the synthesis of Cu-LTA should continue to be explored in future due to the requirement of F^−^ addition in the synthetic process [109,110].

### Other alternative small-pore zeolites

Besides AEI and LTA zeolites, there are also some other small-pore zeolites that should be given attention, as shown in Table S1. The highly crystalline KFI-type zeolite was successfully synthesized via transformation of FAU-type zeolite with Na^+^ and K^+^ ions in the absence of an organic SDA (OSDA) [111]. The Cu ion-exchanged KFI catalyst showed good NH_3_-SCR activity and HTS, which, however, was still inferior to that of Cu-SSZ-13 [111]. Nevertheless, Han *et al.* synthesized high-silica Cu-KFI with Si/Al > 5 by only using K^+^ as a directing agent, which is more sustainable for zeolite synthesis [112]. Importantly, the high-silica Cu-KFI showed excellent HTS and maintained NO*_x_* conversion of ∼57% at 200°C even after HTA at 800°C [112]. Cu-SSZ-50 with the RTH structure, which has 2D channels with aperture sizes of 0.41 nm × 0.38 nm (8MRs) and 0.56 nm × 0.25 nm (distorted 8MRs), also showed comparable NH_3_-SCR activity but relatively low hydrothermal stability [113,114]. Highly active α species and inert β species both existed in Cu-SSZ-50 with high Cu loading, but the α species were easily movable and transferred to more stable sites during HTA [114]. Cu-SSZ-50 can be synthesized at high temperatures in less than 1 hour [115], which gives it significant potential for application in NH_3_-SCR as long as the hydrothermal stability can be improved in future. Martin *et al.* have investigated various cage-based small-pore catalysts, among which Cu-AFX and Cu-ERI were the first to be applied in the NH_3_-SCR reaction. However, although they have similar small-pore structures to Cu-SSZ-13, the SCR activity and HTS still need improvement [116]. Through transformation of FAU zeolite in the presence of an alkali metal-crown ether (AMCE) complex and RHO seeds, Ke *et al.* prepared high-silica RHO zeolite with Si/Al of 7.6. The copper ion-exchanged RHO catalyst showed good NH_3_-SCR performance with relatively high HTS at 800°C [117].

In fact, zeolites with small-pore structures and adequate ion-exchange sites have great potential for utilization as NH_3_-SCR catalysts with high deNO*_x_* efficiency and hydrothermal stability. Researchers have developed many small-pore zeolites in the past several years as discussed above. These zeolites, however, still require comprehensive and systematic in-depth study as well as optimization of physicochemical properties before their practical implementation. Also, it is important that the synthetic method for these developed zeolites is efficient, economical and environmentally friendly. In another aspect, moreover, development of the new type small-pore zeolites with high SCR activity and HTS is still worthwhile based on the design principles proposed above, since there is still considerable room in the small-pore zeolite family for researchers to investigate [118]. Therefore, research should still be devoted to developing and improving small-pore zeolites in the future.

## SUMMARY AND PERSPECTIVE

### General conclusion

This survey provides an easy-to-read and systematic overview of the reported Cu-based small-pore zeolites applied to the NH_3_-SCR reaction in the past decade. Using Cu-SSZ-13 as the main example, we presented an overview of the standard and fast SCR mechanisms, hydrothermal stability, chemical poisoning mechanism (SO_2_, PO_4_^3−^, HCs, alkali and alkaline earth metals), co-cation effects and synthetic methodology. The discovery of the locally homogenous reaction mechanism (SSCR) is a big step forward in the field of catalytic chemistry. This catalytic reaction mechanism will help researchers rationally design catalysts with dynamic active sites in order to achieve high catalytic activity. For actual application of Cu-SSZ-13, the hydrothermal stability and chemical poisoning tolerance should be improved by carefully tuning the properties in the initial synthetic process and/or post-treatment. By precisely controlling the type and amount of co-cation metals, the SCR activity and hydrothermal stability can be improved. Economical and environmentally friendly synthesis routes for the zeolites mainly include the *in situ* method, solvent-free method and template-free method, as well as combinations of these. Each method can achieve sustainable development by using rational and economical raw materials. In addition, the unique properties of other small-pore zeolites, especially AEI- and LTA-type zeolites, were also summarized. Cu-AEI and Cu-LTA zeolites showed hydrothermal stability superior to that of Cu-SSZ-13 under certain conditions, while further breakthroughs still need to be made, such as green and sustainable synthetic methods for these other small-pore zeolites.

### Opportunities

As we pointed out in the introduction section, the typical properties of Cu-exchanged small-pore zeolites match perfectly with the required characteristics of NH_3_-SCR catalysts, resulting in excellent deNO*_x_* efficiency in the NH_3_-SCR reaction. First, the dual-functional Cu^2+^ ion, as both redox and acid site, achieves the limiting case of tight coupling for redox-acid sites in NH_3_-SCR catalysts. Second, the atomic-level dispersal of Cu^2+^ also reaches the limits of high dispersion of active sites in NH_3_-SCR catalysts. Last but not least, the dynamic active sites for the copper-ammonia complex, which is mobile in the zeolite cage, adequately expose them to the reactants. The above three points determine the excellent NH_3_-SCR activity of Cu^2+^-exchanged zeolites. Besides, the shape selectivity of the small-pore structure guarantees its high hydrothermal stability and poisoning resistance. In one aspect, the narrow small pores limit the diffusion of hydrolysed Al(OH)_3_ and accumulation of Cu^2+^, resulting in outstanding hydrothermal stability of framework and active Cu^2+^ sites. In another aspect, some long-chain HCs and large poisoning molecules are prevented from access to the active sites. Therefore, Cu-based small-pore zeolites represent an immense opportunity as efficient and stable NH_3_-SCR catalysts.

In actual application, the consumption of diesel and jet fuel in 2040 will increase 75% compared with that in 2010. Therefore, diesel engines will be irreplaceable as the primary power source for the freight, navigation and marine engine industries and non-road engineering machinery for the foreseeable future, for which saving energy and restraining emissions will be the greatest challenges. Increasingly stringent emission standards are being developed across the world to reduce polluting emissions from diesel engines. In the US, the technological feasibility of achieving an emission limit of 0.02 g/bhp-hr and Low Load Cycle (LLC) limit below 0.075 g/bhp-hr has been demonstrated. In China, highway freight reached ∼40 billion tonnes, accounting for 76.9% of the total freight [5]. The China VI Standards for emissions from diesel-fuelled heavy-duty vehicles will be fully implemented on 1 July 2021. The standard explicitly forbids leakage of V-containing complexes into the atmosphere during the lifetime of vehicles that utilize the V-based SCR catalyst, which indicates that the V-based catalyst will not be applicable for exhaust purification in the future. As an alternative, the development of Cu-based small-pore zeolites with high activity and stability is of vital importance.

### Challenges

Despite the great opportunities offered by Cu-based small-pore zeolites, there are still some challenges to be addressed.

- The chemical poisoning tolerance (SO_2_, P and alkali metals etc.) should be improved. For example, although thermal treatment can recover most of the deNO*_x_* efficiency of S-poisoned zeolite-based catalysts, steady-state SO_2_ poisoning results in a significant decrease due to the formation of ammonium sulphate and copper bisulphite. The addition of other metals as sacrificial components is a possible way to increase the SO_2_ resistance. The small HCs poisoning in the medium temperature range should also be improved in future.
- Although the abnormal FSCR reaction has scarcely any positive effect on the deNO*_x_* efficiency over Cu-based small-pore zeolites, NO_2_ is always present due to the partial oxidation of NO in the DOC part. Therefore, the NO*_x_* reduction pathway in the presence of NO_2_ is still worth investigating. The atomic-level understanding of the abnormal FSCR reaction process is still worthy of study, by suitable experiments accompanied by auxiliary calculations.
- Besides CHA-, LTA- and AEI-type zeolites, development of other small-pore zeolites with suitable elemental composition and pore structure for the NH_3_-SCR reaction, which can be attractive alternative candidates for future NH_3_-SCR catalysts, should be pursued.
- The green and sustainable synthesis of small-pore zeolites is important for actual application. Although some economical and environmentally friendly methods to synthesize Cu-SSZ-13 have been reported, they each have advantages and disadvantages. It is urgently necessary to combine the advantages of these methods, such as by combination of *in situ*, solvent-free and template-free methods. Green and sustainable routes for synthesizing LTA and AEI also need to be addressed in the future. Moreover, the development of ultrafast synthesis methods with various assistive technologies (such as microwave heating or seeded growth) to increase efficiency and reduce the cost is of great importance for industrial application.

## Supplementary Material

nwab010_Supplemental_File
