# Supplementary material for: Selective catalytic reduction of NOx with NH3: opportunities and challenges of Cu-based small-pore zeolites
Source: Natl Sci Rev. 2021 Jan 16;8(10):nwab010. doi: 10.1093/nsr/nwab010 (PMC8566184; doi:10.1093/nsr/nwab010)
Supplement: nwab010_Supplemental_File [file nwab010_supplemental_file.docx]

**Supporting Information**

**Selective catalytic reduction of NO*_x_* with NH_3_: opportunities and challenges of Cu-based small-pore zeolites**

Yulong Shan^a^, Jinpeng Du^a,c^, Yan Zhang^b^, Wenpo Shan^b^, Xiaoyan Shi^a,c^, Yunbo Yu^a,b,c^, Runduo Zhang^d^, Xiangju Meng^e^, Feng-Shou Xiao^e^ and Hong He^a,b,c,^*

^a^State Key Joint Laboratory of Environment Simulation and Pollution Control, Research Center for Eco-Environmental Sciences, Chinese Academy of Sciences, Beijing 100085, China

^b^Center for Excellence in Regional Atmospheric Environment, Institute of Urban Environment, Chinese Academy of Sciences, Xiamen 361021, China

^c^College of Resources and Environment,, University of Chinese Academy of Sciences, Beijing 100049, China

^d^State Key Laboratory of Chemical Resource Engineering, Beijing Key Laboratory of Energy Environmental Catalysis, Beijing University of Chemical Technology, Beijing 100029, China

^e^Key Lab of Applied Chemistry of Zhejiang Province, Department of Chemistry, Zhejiang University, Hangzhou 310007, China

***Corresponding author.** E-mail: honghe@rcees.ac.cn

**Table S1.** SSCR performance of Cu-based small-pore zeolites with different morphologies.

| Zeolite | Structure | Si/Al Cu content (wt.%) | SCR Conditions | DeNOx efficiency (200 ℃/450 ℃) | HTA condition | DeNOx efficiency (200 ℃/450 ℃) | Ref. |
| --- | --- | --- | --- | --- | --- | --- | --- |
| Cu-SSZ-13  (CHA) | 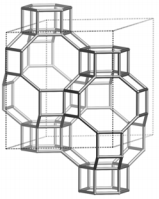 | 5.1  3.8 | [NO]=[NH_3_]=500 ppm, [O_2_]=[H_2_O]=5 vol.%, GHSV = 200,000 h^-1^. | ~100%  ~100% | 10% H_2_O  800 ℃-5 h | ~98%  ~87% | [1] |
| Cu-SSZ-39  (AEI) | 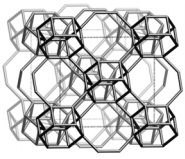 | 6.5  3.4 | [NO]=[NH_3_]=500 ppm, [O_2_]=[H_2_O]=5 vol.%, GHSV = 250,000 h^-1^. | ~87%  ~99% | 10% H_2_O  850 ℃-16 h | ~57%  ~87% | [2] |
| Cu-LTA | 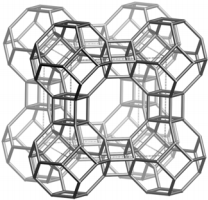 | 16  2.0-3.6  (Cu/Al=0.48) | [NO]=[NH_3_]=500 ppm, [O_2_]=5%, [H_2_O]=5 %, GHSV = 100,000 h^-1^. | ~72%  ~100% | 10% H_2_O  850 ℃-24 h | ~50%  ~95% | [3] |
| Cu-KFI | 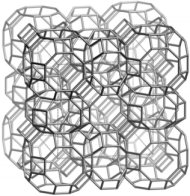 | 5.2  2.8 | [NO]=[NH_3_]=500 ppm, [O_2_]=[H_2_O]=5 vol.%, GHSV = 80,000 h^-1^. | ~98%  ~96% | 10% H_2_O  800 ℃-16 h | ~57%  ~91% | [4] |
| Cu-SSZ-50  (RTH) | 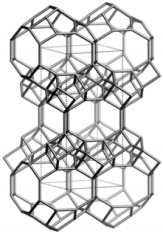 | 8.0  4.4 | [NO]=[NH_3_]=500 ppm, [O_2_]=[H_2_O]=5 vol.%, GHSV = 100,000 h^-1^. | ~98%  ~85% | 10% H_2_O  750 ℃-16 h | ~46%  ~69% | [5] |
| Cu-AFX | 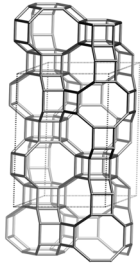 | 5.3  3.1 | [NO]=500 ppm, [NH_3_]=530 ppm, [O_2_]=7%, [H_2_O]=5 vol.%, 40 mg samples with total flow of 300 mL/min | ~56%  ~100% | 10% H_2_O  750 ℃-13 h | ~37%  ~78% | [6] |
| Cu-ERI | 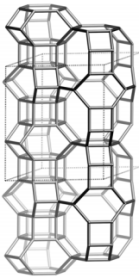 | 5.1  2.2 | [NO]=500 ppm, [NH_3_]=530 ppm, [O_2_]=7%, [H_2_O]=5 vol.%, 40 mg samples with total flow of 300 mL/min | ~48%  ~81% | 10% H_2_O  750 ℃-13 h | ~20%  ~32% | [6] |
| Cu-RHO | 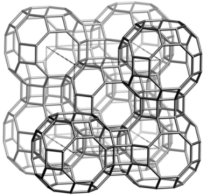 | 7.6  4.0 | [NO]=[NH_3_]=500 ppm, [O_2_]=[H_2_O]=10 vol.%, GHSV = 100,000 h^-1^. | ~53%  ~96% | 10% H_2_O  800 ℃-10 h | ~52%  ~84% | [7] |

1. Shan Y, Du J and Yu Y *et al*. Precise control of post-treatment significantly increases hydrothermal stability of in-situ synthesized cu-zeolites for NH_3_-SCR reaction. *Appl Catal B* 2020; **266**: 118655.

2. Shan Y, Shan W and Shi X et al. A comparative study of the activity and hydrothermal stability of Al-rich Cu-SSZ-39 and Cu-SSZ-13. *Appl Catal B* 2020; **264**: 118511.

3. Ryu T, Ahn NH and Seo S et al. Fully Copper-Exchanged High-Silica LTA Zeolites as Unrivaled Hydrothermally Stable NH_3_-SCR Catalysts. *Angew Chem Int Ed* 2017; **56**: 3256-60.

4. Han S, Tang X and Wang L et al Potassium-directed sustainable synthesis of new high silica small-pore zeolite with KFI structure (ZJM-7) as an efficient catalyst for NH_3_-SCR reaction. *Appl Catal B* 2021; **281**: 119480.

5. Shan Y, Shi X and Du J *et al*. Cu-exchanged RTH-type zeolites for NH_3_-selective catalytic reduction of NO*_x_*: Cu distribution and hydrothermal stability. *Catal Sci Technol* 2019; **9**: 106-15.

6. Martín N, Paris C and Vennestrøm PNR *et al*. Cage-based small-pore catalysts for NH_3_-SCR prepared by combining bulky organic structure directing agents with modified zeolites as reagents. *Appl Catal B* 2017; **217**: 125-36.

7. Ke Q, Sun T and Cheng H *et al*. Accelerated Construction of High-Silica RHO and CHA Zeolites via Interzeolite Transformation and Their NH_3_–SCR Performances after Copper Exchange. *Ind Eng Chem Res* 2018; **57**: 16763-71.
